# Supplementary material for: Numerical analysis of the impact of cytoskeletal actin filament density alterations onto the diffusive vesicle-mediated cell transport
Source: PLoS Comput Biol. 2021 May 3;17(5):e1008784. doi: 10.1371/journal.pcbi.1008784 (PMC8130967; doi:10.1371/journal.pcbi.1008784)
Supplement: S1 Appendix — In this appendix, we provide some details on the implementation of a finite element method to find unique solutions of the diffusion problem and the cell problem, as introduced in Eqs (2) to (4). (PDF) [file pcbi.1008784.s002.pdf]

Supporting information

S1 Appendix. Finite element implementation

In this appendix, we provide some details on the implementation of a finite element method to find unique solutions of the diffusion problem and the cell problem, as introduced in Eqs (2) to (4) of the main article.

The diffusion problem

By means of the Dirichlet lift, $u^\varepsilon = \hat{u}^\varepsilon + g_\mathrm{D}$, the variational (or weak) formulation of Eq (2) in the main article is used to find $\hat{u}^\varepsilon(t) \in H^1_D(\Omega)$ for $t > t_0$ such that

$$\int_\Omega \frac{\partial}{\partial t}\hat{u}^\varepsilon(t)\, v \,\mathrm{d}\mathbf{x} + \underbrace{\int_\Omega \mathbf{D}^\varepsilon \nabla \hat{u}^\varepsilon(t) \cdot \nabla v \,\mathrm{d}\mathbf{x}}_{=:B^\varepsilon(\hat{u}^\varepsilon(t),v)} = \int_\Omega f v \,\mathrm{d}\mathbf{x} + \int_{\partial\Omega_N} g_\mathrm{N} v \,\mathrm{d}s_\mathbf{x} \tag{S1.1}$$
$$- \int_\Omega \mathbf{D}^\varepsilon \nabla g_\mathrm{D} \cdot \nabla v \,\mathrm{d}\mathbf{x} =: l^\varepsilon(v), \forall v \in H^1_D(\Omega).$$

Using the standard Galerkin finite element method in space together with the backward Euler method in time, i.e. $\partial \hat{u}^\varepsilon(t_n)/\partial t = (\hat{u}^\varepsilon(t_n) - \hat{u}^\varepsilon(t_{n-1}))/\Delta t$, where $t_n = t_0 + n\Delta t$ with $n \in \mathbb{N}^+$ and the time interval of interest split into equally sized subintervalls $\Delta t > 0$, the corresponding discretized formulation of Eq (S1.1) reads:

Find $\hat{u}^\varepsilon_H(t_n) \in V^p_D(\Omega, \mathcal{T}_H)$ for $t_n > t_0$ such that

$$\int_\Omega \hat{u}^\varepsilon_H(t_n)\, v_H \,\mathrm{d}\mathbf{x} + \Delta t\, B^\varepsilon(\hat{u}^\varepsilon_H(t_n), v_H) = \int_\Omega \hat{u}^\varepsilon_H(t_{n-1})\, v_H \,\mathrm{d}\mathbf{x} + \Delta t\, l^\varepsilon(v_H), \tag{S1.2}$$
$$\forall v_H \in V^p_D(\Omega, \mathcal{T}_H),$$

where $V^p_D(\Omega, \mathcal{T}_H) = \left\{ v_H \in H^1_D(\Omega) : v_H|_K \in \mathcal{R}^p(K), \forall K \in \mathcal{T}_H \right\}$ is a finite dimensional subspace of $H^1_D(\Omega)$ with a suitable space of polynomials $\mathcal{R}^p(K)$ of order p, and $\mathcal{T}_H$ denotes the regular triangulation of Ω into finite elements K. Edges of the finite elements that belong to the boundary $\partial\Omega$ are denoted by $E \subset \partial\Omega$. The discretized weak formulation of the problem in Eq (S1.2) leads to the linear system

$$(\mathbf{M} + \Delta t \mathbf{A})\, \mathbf{u}^\varepsilon_H(t_n) = \mathbf{b} + \mathbf{M}\mathbf{u}^\varepsilon_H(t_{n-1}), \tag{S1.3}$$

where $\mathbf{u}^\varepsilon_H(t_n) \in \mathbb{R}^N$ contains the unknown nodal solutions as well as the incorporated Dirichlet conditions, $\mathbf{u}^\varepsilon_H(t_{n-1}) \in \mathbb{R}^N$ contains the already known nodal solutions from the previous time step, $\mathbf{M} \in \mathbb{R}^{N\times N}$ and $\mathbf{A} \in \mathbb{R}^{N\times N}$ are the global mass and stiffness matrices, respectively, defined as

$$M_{jk} = \sum_{K\in\mathcal{T}_H} \int_K \eta_j \eta_k \,\mathrm{d}\mathbf{x}, \qquad A_{jk} = \sum_{K\in\mathcal{T}_H} \int_K \mathbf{D}^\varepsilon \nabla \eta_j \cdot \nabla \eta_k \,\mathrm{d}\mathbf{x}, \tag{S1.4}$$

with $\{\eta_k\}_{k=1}^N$ being the basis functions of the space $V^p_D(\Omega, \mathcal{T}_H)$ and N is the number of degrees of freedom. The vector $\mathbf{b} \in \mathbb{R}^N$ with entries

$$b_j = \sum_{K\in\mathcal{T}_H} \int_K f \eta_j \,\mathrm{d}\mathbf{x} + \sum_{E\subset\partial\Omega_N} \int_E g_\mathrm{N} \eta_j \,\mathrm{d}s_\mathbf{x} - \sum_{k=1}^{N_D} g_{D_k} \sum_{K\in\mathcal{T}_H} \int_K \mathbf{D}^\varepsilon \nabla \eta_j \cdot \nabla \eta_k \,\mathrm{d}\mathbf{x} \tag{S1.5}$$

corresponds to $l^{\varepsilon}(v_H)$ in Eq (S1.2), where $k = \{1, \ldots, N_D\}$ indicates the summation over all nodes that belong to $\partial\Omega_D$. The integrals in Eqs (S1.4) and (S1.5) are solved by means of numerical integration, e.g., using a Gaussian quadrature rule. The matrices $\mathbf{M}$ and $\mathbf{A}$ are sparse, symmetric and positive definite, such that Eq (S1.3) has exactly one solution.

The cell problem

The weak discretized form of the j cell problems, as introduced in Eq (4) of the main article, reads:

Find $\chi_h^j \in S_{\text{per}}^q(K_\delta, \mathcal{T}_h)$ such that

$$\int_{K_\delta} \mathbf{D}^{\varepsilon}(\mathbf{x}) \nabla \chi_h^j \cdot \nabla v_h \mathrm{d}\mathbf{x} = \int_{K_\delta} \mathbf{D}^{\varepsilon}(\mathbf{x}) \, \mathbf{e}_j \cdot \nabla v_h \mathrm{d}\mathbf{x}, \quad \forall v_h \in S_{\text{per}}^q(K_\delta, \mathcal{T}_h), \tag{S1.6}$$

where $S_{\text{per}}^q(K_\delta, \mathcal{T}_h) = \left\{ v_h \in W_{\text{per}}^1(K_\delta) : v_h|_T \in \mathcal{R}^q(T), \forall T \in \mathcal{T}_h \right\}$ is a finite dimensional subspace of $W_{\text{per}}^1(K_\delta) = \{v \in H_{\text{per}}^1(K_\delta), \int_{K_\delta} v \, \mathrm{d}\mathbf{x} = 0\}$ with a suitable space of polynomials $\mathcal{R}^q(T)$ of order q, and $\mathcal{T}_h$ denotes the regular triangulation of K_δ into finite elements T, see Fig 4C in the main article. The space $W_{\text{per}}^1(K_\delta)$ determines the boundary conditions used for computing the microfunctions χ_h^j, and $H_{\text{per}}^1(K_\delta)$ is defined as the closure of the subset of $\mathcal{C}^\infty(\mathbb{R}^n)$ of periodic functions in K_δ. In order to incorporate the periodic boundary conditions as well as the zero average constraint, the discretized weak formulation of the cell problem (see Eq (S1.6)) was rewritten as a saddle point problem of the form

$$\begin{bmatrix} \mathbf{A} & \mathbf{C}^{\mathrm{T}} \\ \mathbf{C} & \mathbf{0} \end{bmatrix} \begin{bmatrix} \boldsymbol{\chi}_h^j \\ \boldsymbol{\lambda} \end{bmatrix} = \begin{bmatrix} \mathbf{f}^j \\ \mathbf{0} \end{bmatrix}, \tag{S1.7}$$

where $\mathbf{A} \in \mathbb{R}^{N_{\text{dof}} \times N_{\text{dof}}}$ is the stiffness matrix corresponding to the left-hand side of Eq (S1.6), as defined in Eq (S1.4) with N_{dof} being the number of microscopic degrees of freedom. The constraint matrix $\mathbf{C} \in \mathbb{R}^{N_{\text{constr}} \times N_{\text{dof}}}$ with N_{constr} being the number of microscopic constraints has the form

$$[\mathbf{C}] = \begin{bmatrix} c_1 \ldots c_{N_{\text{dof}}} \\ \widetilde{\mathbf{C}} \end{bmatrix}, \qquad \text{with} \qquad c_i = \sum_{T \in \mathcal{T}_h} \int_T \eta_i \, \mathrm{d}\mathbf{x}, \tag{S1.8}$$

where the first row in $\mathbf{C}$ corresponds to the zero average condition of $\chi^j(\mathbf{x})$, as introduced in Eq (4) of the main article. The periodicity constraint of $\chi^j(\mathbf{x})$ is considered by means of the matrix $\widetilde{\mathbf{C}} \in \mathbb{R}^{(N_{\text{constr}}-1) \times N_{\text{dof}}}$. Therefore, the $i = \{1, \ldots, (N_{\text{constr}} - 1)\}$ non-redundant couples at opposite sides of the sampling domain K_δ and their node numbers, denoted by $p(i)$ and $p'(i)$, need to be identified. Hence, the matrix $\widetilde{\mathbf{C}}$ has the entry $\widetilde{C}_{ij} = 1$ if $j = p(i)$, $\widetilde{C}_{ij} = -1$ if $j = p'(i)$ and $\widetilde{C}_{ij} = 0$ otherwise. Furthermore, $\boldsymbol{\chi}_h^j \in \mathbb{R}^{N_{\text{dof}}}$ contains the nodal solutions of the cell problem j, $\boldsymbol{\lambda} = [\lambda_1, \ldots, \lambda_{N_{\text{constr}}}]^{\mathrm{T}} \in \mathbb{R}^{N_{\text{constr}}}$ is a vector consisting of the Lagrange multipliers λ_i, and $\mathbf{f}^j \in \mathbb{R}^{N_{\text{dof}}}$ corresponds to the right-hand side of the cell problem j, as introduced in Eq (S1.6), with the entries

$$f_k^j = \sum_{T \in \mathcal{T}_h} \int_T \mathbf{D}^{\varepsilon} \mathbf{e}_j \cdot \nabla \eta_k \, \mathrm{d}\mathbf{x}. \tag{S1.9}$$
