# Supplementary material for: Numerical analysis of the impact of cytoskeletal actin filament density alterations onto the diffusive vesicle-mediated cell transport
Source: PLoS Comput Biol. 2021 May 3;17(5):e1008784. doi: 10.1371/journal.pcbi.1008784 (PMC8130967; doi:10.1371/journal.pcbi.1008784)
Supplement: S2 Appendix — This appendix presents detailed descriptions and results of the validation and verification process regarding the introduced multiscale model for the diffusive vesicle transport based on the concept of homogenization. (PDF) [file pcbi.1008784.s003.pdf]

Supporting information

S2 Appendix. Validation and verification

This appendix presents detailed descriptions and results of the validation and verification process regarding the introduced multiscale model for the diffusive vesicle transport based on the concept of homogenization. As suggested by Novak et al. [1, 2], validation was performed by comparing the effective diffusion coefficients D^0_{ij}, computed by the proposed multiscale method at the sampling domain K_δ, with the coefficients $\widetilde{D}^0_{ij}$, obtained by a Monte Carlo simulation of vesicles undergoing random walks on the same domain K_δ. The correct finite element implementation of the multiscale method, i.e. the micro- and macroscopic solvers and their interface, was verified using a one-dimensional elliptic second-order partial differential equation with an oscillatory coefficient originating from a fine-scale ε, for which an analytical solution can be computed.

Validation: Monte Carlo simulation versus homogenization strategy

In Monte Carlo simulations of vesicles undergoing random walks in the cytosol, the current position $\mathbf{x}_i(t_j) \in \mathbb{R}^d$ of the vesicle $i = \{1, \ldots, N_{\text{part}}\}$ at time t_j is computed as

$$\mathbf{x}_i(t_j) = \mathbf{x}_i(t_{j-1}) + \mathbf{m}_i(t_j)\sqrt{2dD_0\Delta t}, \tag{S2.1}$$

where $\mathbf{x}_i(t_{j-1})$ is the previous position of the vesicle, $\mathbf{m}_i(t_j) \in [-1, 1]^d$, with $|\mathbf{m}_i| = 1$, is a random unit vector (drawn from a uniform distribution for each vesicle i and at each point in time t_j), $d = \{1, 2, 3\}$, is the dimension of the domain, $D_0 \in \mathbb{R}$ is the diffusion coefficient of the cytosol (i.e. only the free space) which is assumed to be isotropic, and Δt is a fixed time step. If a vesicle reaches an inaccessible domain due to the finite vesicle size, the updated position $\mathbf{x}_i(t_j)$ is rejected and a new random vector $\mathbf{m}_i(t_j)$ is drawn until the vesicle has found an accessible domain. The resulting effective diffusion tensor $\widetilde{\mathbf{D}}^0 \in \mathbb{R}^{d\times d}$ is calculated from the covariance matrix $\mathbf{\Sigma}(\mathbf{X}) \in \mathbb{R}^{d\times d}$ of the displacement matrix $\mathbf{X} = \left[\mathbf{x}_1(t_{\max}) - \mathbf{x}_1(t_0), \ldots, \mathbf{x}_{N_{\text{part}}}(t_{\max}) - \mathbf{x}_{N_{\text{part}}}(t_0)\right]^{\mathrm{T}} \in \mathbb{R}^{N_{\text{part}}\times d}$ normalized by the diffusion time $t_{\max}$, i.e.

$$\widetilde{\mathbf{D}}^0 = \frac{\mathbf{\Sigma}(\mathbf{X})}{2t_{\max}} \quad \text{with} \quad \mathbf{\Sigma}(\mathbf{X}) = \frac{1}{N_{\text{part}} - 1}\left(\mathbf{X} - \frac{1}{N_{\text{part}}}\tilde{\mathbf{I}}\mathbf{X}\right)^{\mathrm{T}}\left(\mathbf{X} - \frac{1}{N_{\text{part}}}\tilde{\mathbf{I}}\mathbf{X}\right), \tag{S2.2}$$

where $\tilde{\mathbf{I}} \in \mathbb{R}^{N_{\text{part}}\times N_{\text{part}}}$ is a matrix of ones. Note, the larger $t_{\max}$ and N_{part} the more accurate $\widetilde{\mathbf{D}}^0$ gets.

The aim of this numerical example was to validate the effective diffusion coefficients computed via the proposed homogenization method by means of a Monte Carlo simulation. Therefore, a 2D square unit cell (sampling domain) with edge length $L = 25\,\mu$m was generated containing one circular void (obstacle) with a radius r located at the center of the unit cell. An isotropic diffusion tensor $\mathbf{D}^\varepsilon = D_0\mathbf{I}$ with $D_0 = 1\,\mu\text{m}^2/\text{s}$ was defined at the domain outside the void. Hence, for this axisymmetric unit cell, an isotropic effective diffusion tensor $\mathbf{D}^0 = D^0\mathbf{I}$ was expected. To study the effect of different inaccessible area fractions $\phi = r^2\pi/L^2$ on the effective diffusion coefficient D^0, the radius r of the void was varied between 0 and $L/2$. For the computation of the homogenized diffusion tensor, the sampling domain was discretized using triangular linear finite elements. A mesh refinement study was performed to ensure that converged effective diffusion tensors were obtained. The Monte Carlo simulations were performed

on a domain that contained 6×6 unit cells, placed next to each other. In total, 10 000 vesicles were positioned at the center of the 36 unit cells. All vesicles were treated to be independent. Hence, interactions between vesicles were not considered. The size of the time step was set to $\Delta t = 0.01\,\mathrm{s}$. In total 10 Monte Carlo simulations for each inaccessible area fraction ϕ were performed and the resulting mean behavior was analyzed.

Fig S2.1A shows the time dependency of the magnitude of the mean-squared displacement $|\langle \mathbf{x}^2(t) \rangle| \propto t^\alpha$ with $\alpha \in \mathbb{R}$ for different inaccessible area fractions ϕ. Thereby, $\phi = 0$ denotes an unobstructed diffusion of the vesicles in the cytosol with diffusion coefficient D_0. With increasing inaccessible area fractions ϕ the curves exhibit three distinct diffusion regimes: (i) an unobstructed (normal) diffusion ($\alpha = 1$) on length scales less than the distance between neighboring obstacles; (ii) an anomalous diffusion ($\alpha < 1$) on the length scale where vesicles start to collide with obstacles; (iii) a normal diffusion ($\alpha = 1$) on a larger length scale characterized by an effective diffusion coefficient $\widetilde{D}^0$. However, for large values of ϕ the vesicles were trapped by the obstacles and $|\langle \mathbf{x}^2(t) \rangle|$ was no longer proportional to t. Fig S2.1B compares the diffusion coefficients $\widetilde{D}^0$ obtained by the Monte Carlo simulations using Eq (S2.2) with the diffusion coefficients D^0 obtained by the proposed homogenization strategy for different inaccessible area fractions ϕ, both normalized by the diffusion coefficient of the cytosol D_0. It can be seen that for $0 \leq \phi \leq 0.6$ the relative effective diffusion coefficient is decreasing almost proportional with increasing ϕ. For $\phi > 0.6$ the relative effective diffusion coefficient decreases significantly, indicating a percolation limit of $\phi_c \approx 0.8$. Furthermore, the values computed by the homogenization strategy compare well with the values obtained by the Monte Carlo simulations, which is in good agreement with Novak et al. [1, 2]. However, the estimation of the effective diffusion coefficients using the homogenization strategy takes only a couple of minutes, while the Monte Carlo simulation takes several hours, indicating that the homogenization method is much more efficient computationally.

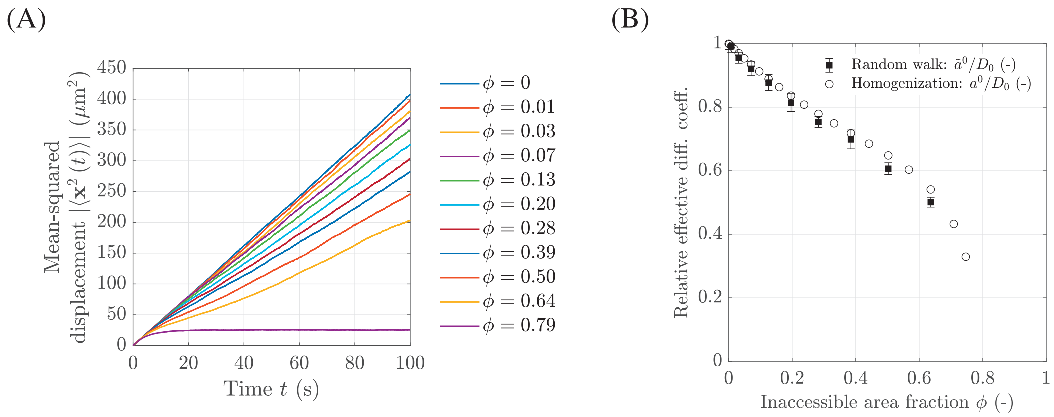

Fig S2.1. Monte Carlo simulations (random walk). (A) Magnitude of the mean-squared displacement $|\langle \mathbf{x}^2(t) \rangle|$ as a function of time for different inaccessible area fractions ϕ. (B) Comparison of the relative effective diffusion coefficient estimates as a function of the inaccessible area fraction ϕ obtained by Monte Carlo simulations (filled squares with error bars denoting mean $\pm$ SD) and by the proposed homogenization strategy using the finite element method (circles). Data used in this figure can be found in the supporting information S1 Data.

Verification: solving a one-dimensional elliptic partial differential equation with an oscillatory coefficient originating from a fine-scale

The correct implementation of the multiscale FE code was carefully analyzed by means of a simple one-dimensional (1D) problem for which a closed analytical solution exists. This example primarily aims to study the effects of different macroscopic and microscopic mesh sizes (denoted by H and h) on the convergence behavior of the error between the analytical and the numerical solution. Therefore, we consider the following elliptic problem

$$-\frac{\mathrm{d}}{\mathrm{d}x}\left(D^{\varepsilon}(x)\frac{\mathrm{d}}{\mathrm{d}x}u^{\varepsilon}(x)\right) = 1, \quad \forall x \in [0,1], \qquad u^{\varepsilon}(0) = u^{\varepsilon}(1) = 0, \tag{S2.3}$$

where an oscillatory coefficient is given by $D^{\varepsilon}(x) = 1/\left[2+\cos\left(2\pi x/\varepsilon\right)\right]$, $\forall x \in (-\infty, +\infty)$, with a period length $\varepsilon > 0$, see Fig S2.2A.

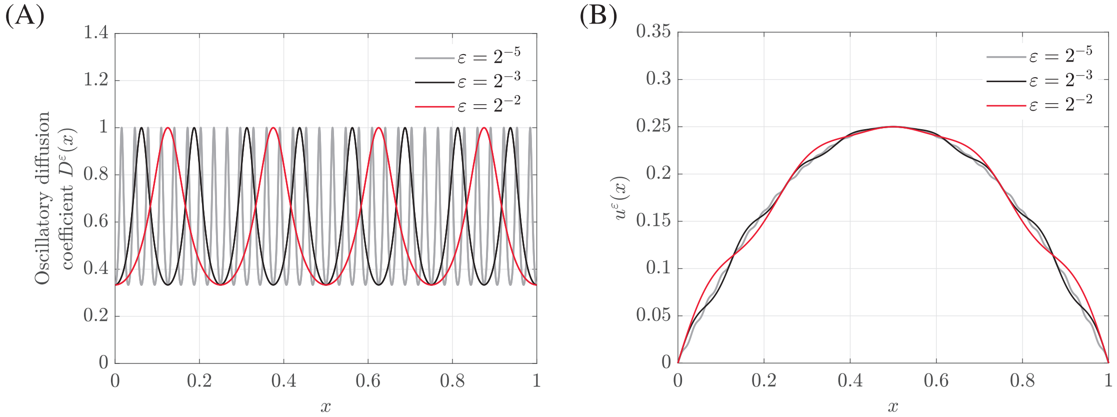

Fig S2.2. The effect of three different values of the periodic length ε on: (A) the oscillatory coefficient $D^{\varepsilon}(x)$; (B) the analytical solution $u^{\varepsilon}(x)$ of the boundary-value problem, as given in Eq (S2.4). Data used in this figure can be found in the supporting information S1 Data.

For numerical verification purposes, we restrict ourselves to a rather simple mathematical problem with limited relevance to the vesicle-mediated cell transport and physical meaning but being utterly useful to show that the proposed multiscale finite element method was implemented correctly. Nevertheless, the oscillatory coefficient $D^{\varepsilon}(x)$ could be interpreted as the rapidly changing diffusion properties in a perfectly periodic medium. The smaller ε gets the smaller the heterogeneities become so that the medium starts to take on the appearance of a homogeneous medium, and hence it would behave like a homogeneous medium on the macroscopic scale. From a physical point of view, Eq (S2.3) denotes the steady-state version of the diffusion problem, as introduced in Eq (2) of the main article, where $u^{\varepsilon}(x)$ would be the steady-state vesicle concentration (per unit volume), and the source term indicates the production of vesicles inside the cell domain. Furthermore, it is assumed that no vesicles are found at the left and right boundaries of the 1D cell domain Ω. However, the reader should not overstate the physical interpretation of this mathematical verification example. The corresponding unique analytical solution of the problem is given by

$$u^{\varepsilon}(x) = -x^2 + x + \varepsilon\left[\frac{1}{4\pi}\sin\left(2\pi\frac{x}{\varepsilon}\right) - \frac{1}{2\pi}x\sin\left(2\pi\frac{x}{\varepsilon}\right) - \frac{\varepsilon}{4\pi^2}\cos\left(2\pi\frac{x}{\varepsilon}\right) + \frac{\varepsilon}{4\pi^2}\right], \tag{S2.4}$$

which consists of a macroscopic part being independent of ε, and a strongly oscillatory part. Note that the use of a standard P1-FEM requires a mesh size $H \ll \varepsilon$ to obtain reasonable approximations, even on the macroscopic scale of interest. Hence for small values of ε, the computational cost becomes unacceptable even for this simple 1D problem. From Eq (S2.4) it can be seen that the oscillatory solution $u^{\varepsilon}(x)$ converges to a homogenized solution $u^0(x) = -x^2 + x$ as $\varepsilon \to 0$, compare with Fig S2.2B.

To quantify the effective coefficient $D^0(\hat{x}_{\text{ip}})$ at a given integration point $\hat{x}_{\text{ip}}$ within a macroscopic finite element, the cell problem, as introduced in Eq (4) of the main article, needs to be solved, i.e.

$$\begin{aligned} &\frac{\mathrm{d}}{\mathrm{d}x}\left[D^{\varepsilon}(x)\left(1-\frac{\mathrm{d}}{\mathrm{d}x}\chi^1(x)\right)\right]=0, \quad \forall x \in \left[\hat{x}_{\text{ip}}-\frac{\delta}{2}, \hat{x}_{\text{ip}}+\frac{\delta}{2}\right], \\ &\chi^1\left(\hat{x}_{\text{ip}}-\delta/2\right)=\chi^1\left(\hat{x}_{\text{ip}}+\delta/2\right), \\ &\int_{\hat{x}_{\text{ip}}-\delta/2}^{\hat{x}_{\text{ip}}+\delta/2}\chi^1(x)\,\mathrm{d}x=0, \end{aligned} \tag{S2.5}$$

where δ denotes the size of the sampling domain. Integrating Eq (S2.5) twice and applying the periodicity constraint as well as the zero-average constraint yields the unique analytical solution $\chi^1(x)$ and its first derivative $\mathrm{d}\chi^1(x)/\mathrm{d}x$, respectively, i.e.

$$\chi^1(x)=x-c_1\left[2x+\sin\left(2\pi\frac{x}{\varepsilon}\right)\frac{\varepsilon}{2\pi}\right]+c_2, \qquad \frac{\mathrm{d}}{\mathrm{d}x}\chi^1(x)=1-c_1\left[2+\cos\left(2\pi\frac{x}{\varepsilon}\right)\right], \tag{S2.6}$$

with the integration constants

$$\begin{aligned} c_1 &= \left\{2+\frac{1}{2\pi}\frac{\varepsilon}{\delta}\left[\sin\left(\frac{2\pi}{\varepsilon}\left(\hat{x}_{\text{ip}}+\frac{\delta}{2}\right)\right)-\sin\left(\frac{2\pi}{\varepsilon}\left(\hat{x}_{\text{ip}}-\frac{\delta}{2}\right)\right)\right]\right\}^{-1} \\ &= \left\{2+\frac{1}{\pi}\frac{\varepsilon}{\delta}\left[\sin\left(\pi\frac{\delta}{\varepsilon}\right)\cos\left(\frac{2\pi}{\varepsilon}\hat{x}_{\text{ip}}\right)\right]\right\}^{-1}, \\ c_2 &= \hat{x}_{\text{ip}}\left(2c_1-1\right)+c_1\frac{\varepsilon^2}{4\pi^2}\frac{1}{\delta}\left[\cos\left(2\pi\left(\hat{x}_{\text{ip}}-\frac{\delta}{2}\right)\right)-\cos\left(2\pi\left(\hat{x}_{\text{ip}}+\frac{\delta}{2}\right)\right)\right]. \end{aligned} \tag{S2.7}$$

According to Eq (5) in the main article, the analytically computed effective diffusion coefficient is given by

$$\begin{aligned} D^0\left(\hat{x}_{\text{ip}}\right) &= \frac{1}{\delta}\int_{\hat{x}_{\text{ip}}-\delta/2}^{\hat{x}_{\text{ip}}+\delta/2} D^{\varepsilon}(x)\left(1-\frac{\mathrm{d}}{\mathrm{d}x}\chi^1(x)\right)\mathrm{d}x \\ &= \frac{1}{\delta}\int_{\hat{x}_{\text{ip}}-\delta/2}^{\hat{x}_{\text{ip}}+\delta/2}\frac{1}{2+\cos(2\pi x/\varepsilon)}c_1\left[2+\cos(2\pi x/\varepsilon]\right)\mathrm{d}x=c_1, \end{aligned} \tag{S2.8}$$

using the solution of the cell problem, see Eqs (S2.5) to (S2.7). From Eqs (S2.7) and (S2.8) it can be seen that the effective coefficient $D^0(\hat{x}_{\text{ip}}) = c_1$ depends on the size of the sampling domain δ and the period length ε as well as the integration point $\hat{x}_{\text{ip}}$, and therefore indirectly on the macroscopic mesh size H.

For the given steady-state problem, see Eq (S2.3), and by using linear shape functions for the $N_{\text{mac}} = 1/H$ macroscopic finite elements, the two integration points $\hat{x}^k_{\text{ip},1}$ and $\hat{x}^k_{\text{ip},2}$ of the finite element k can be expressed in terms of the macroscopic mesh size H, i.e.

$$\hat{x}^k_{\text{ip},m}=\frac{H}{2}\left((-1)^m/\sqrt{3}+2k-1\right), \tag{S2.9}$$

with $k = \{1, 2, \ldots, N_{\text{mac}}\}$, and $m = \{1, 2\}$. Hence, the effective diffusion coefficient D^0 becomes

$$D^0(\varepsilon, \delta, H, m, k) = \left\{2 + \frac{1}{\pi}\frac{\varepsilon}{\delta}\left[\sin\left(\pi\frac{\delta}{\varepsilon}\right)\cos\left(\pi\frac{H}{\varepsilon}\left(\frac{(-1)^m}{\sqrt{3}} + 2k - 1\right)\right)\right]\right\}^{-1}$$
$$= \left\{2 + \frac{1}{\pi}\frac{1}{\tilde{x}}\left[\sin(\pi\tilde{x})\cos\left(\pi\tilde{y}\left(\frac{(-1)^m}{\sqrt{3}} + 2k - 1\right)\right)\right]\right\}^{-1}$$
$$= D^0(\tilde{x}, \tilde{y}, m, k), \tag{S2.10}$$

with the fractions $\tilde{x} = \delta/\varepsilon$ and $\tilde{y} = H/\varepsilon$. Fig S2.3 highlights how different values of $\tilde{x}$ and $\tilde{y}$ affect the homogenized effective coefficient D^0, evaluated at the first integration point of the first element ($m = k = 1$), which is known as the cell resonance phenomenon. From Eq (S2.10) it can be seen that the correct effective coefficient $D^0 = 1/2$ can only be achieved if either $\tilde{x} \in \mathbb{N}^+, \forall \tilde{y} \in \mathbb{R}$, or if

$$\tilde{y} = \frac{j - 1/2}{(-1)^m/\sqrt{3} + 2k - 1}, \quad \forall j \in \mathbb{N}^+, \forall \tilde{x} \in \mathbb{R}, \tag{S2.11}$$

identified by the black contour lines in Fig S2.3.

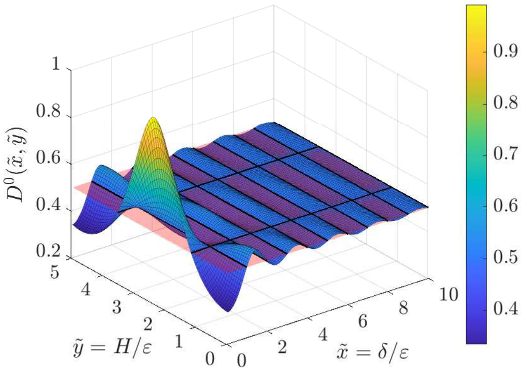

Fig S2.3. Resonance phenomenon of the homogenized diffusion coefficient. The effect of different values of $\tilde{x} = \delta/\varepsilon$ and $\tilde{y} = H/\varepsilon$ on the homogenized coefficient $D^0(\tilde{x}, \tilde{y})$, as defined in Eq (S2.10) for $m = k = 1$. Black lines indicate contour lines where $D^0(\tilde{x}, \tilde{y}) = 0.5$. The red plane is lying parallel to the $(\tilde{x}, \tilde{y})$ plane at a level of 0.5 indicating the oscillatory behavior of $D^0(\tilde{x}, \tilde{y})$ around the correct analytical value of 0.5. Data used in this figure can be found in the supporting information S1 Data.

Finally, solving the homogenized elliptic problem

$$-\frac{\mathrm{d}}{\mathrm{d}x}\left(D^0\frac{\mathrm{d}}{\mathrm{d}x}u^0(x)\right) = 1, \quad \forall x \in [0, 1], \qquad u^0(0) = u^0(1) = 0, \tag{S2.12}$$

by means of the effective coefficient $D^0 = 1/2$, as computed in Eq (S2.8) for $\delta = \varepsilon$, it can be seen that the homogenized solution $u^0(x) = -x^2 + x$ corresponds to the macroscopic part of the solution $u^\varepsilon(x)$, see Eq (S2.4). Since the given coefficient $D^\varepsilon(x)$ is periodic, the estimation of the effective coefficient $D^0(x) = D^0$ at one single integration point is sufficient to obtain the homogenized solution $u^0(x)$ if the sampling domain size δ is set to the period length ε of the oscillatory coefficient $D^\varepsilon(x)$.

Fig S2.4 compares the obtained analytical as well as the numerical solutions of the cell problem $\chi^1(x)$ and the macroscopic (homogenized) solution $u^0(x)$, respectively. Furthermore, effects of microscopic and macroscopic mesh refinements on the convergence behavior of the cell problem solution and the macroscopic solutions of the given steady-state problem are analyzed. While Fig S2.4B depicts a perfectly quadratic convergence behavior of the microscopic error for a decreasing microscopic mesh size h, Fig S2.4D highlights the remanent macroscopic error obtained for a given macroscopic mesh size H and a constantly decreasing microscopic mesh size h. This macroscopic error can only be reduced by decreasing the macroscopic mesh size.

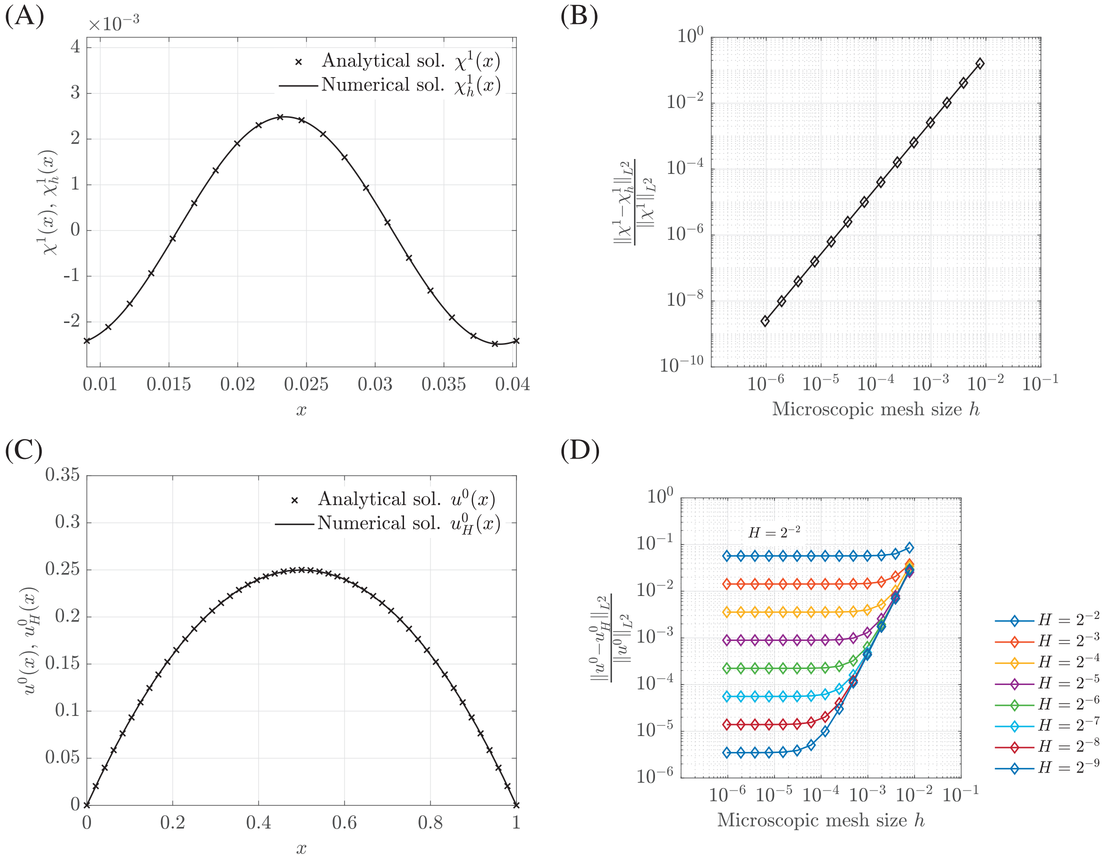

Fig S2.4. Results of the multiscale FE analyzes for a cell size $\delta = \varepsilon = 2^{-5}$. (A) FE solution $\chi_h^1(x)$ in comparison to the analytical solution $\chi^1(x)$ of the cell problem, as given in Eq (S2.5), at the macroscopic integration point $\hat{x}_{\text{ip}} = 0.0246$ for a microscopic mesh size $h = \varepsilon/2^{15} \ll \varepsilon$ and a macroscopic mesh size $H = \varepsilon$. (B) Evolution of the relative L^2-norm of the error between the analytical solution $\chi^1(x)$ and the FE solution $\chi_h^1(x)$ for a decreasing microscopic mesh size h. (C) FE solution $u_H^0(x)$ in comparison to the analytical solution $u^0(x)$ for the same discretization as described in (A). (D) Relative L^2-norm of the error between the analytical solution u^0 and the FE solution u_H^0 as a function of the microscopic mesh size h. Each curve corresponds to a constant macroscopic mesh size H. Data used in this figure can be found in the supporting information S1 Data.

The oscillatory character of the solution can be recovered by using the first order asymptotic

expansion, as introduced in Eq (6) of the main article, resulting in

$$\begin{aligned}\tilde{u}^{\varepsilon}(x) &= u^{0}(x) - \chi^{1}(x)\frac{\mathrm{d}}{\mathrm{d}x}u^{0}(x) \\ &= -x^{2} + x + \varepsilon\left[\frac{1}{4\pi}\sin\left(2\pi\frac{x}{\varepsilon}\right) - \frac{1}{2\pi}x\sin\left(2\pi\frac{x}{\varepsilon}\right)\right] \approx u^{\varepsilon}(x),\end{aligned} \tag{S2.13}$$

as can be seen in Fig S2.5.

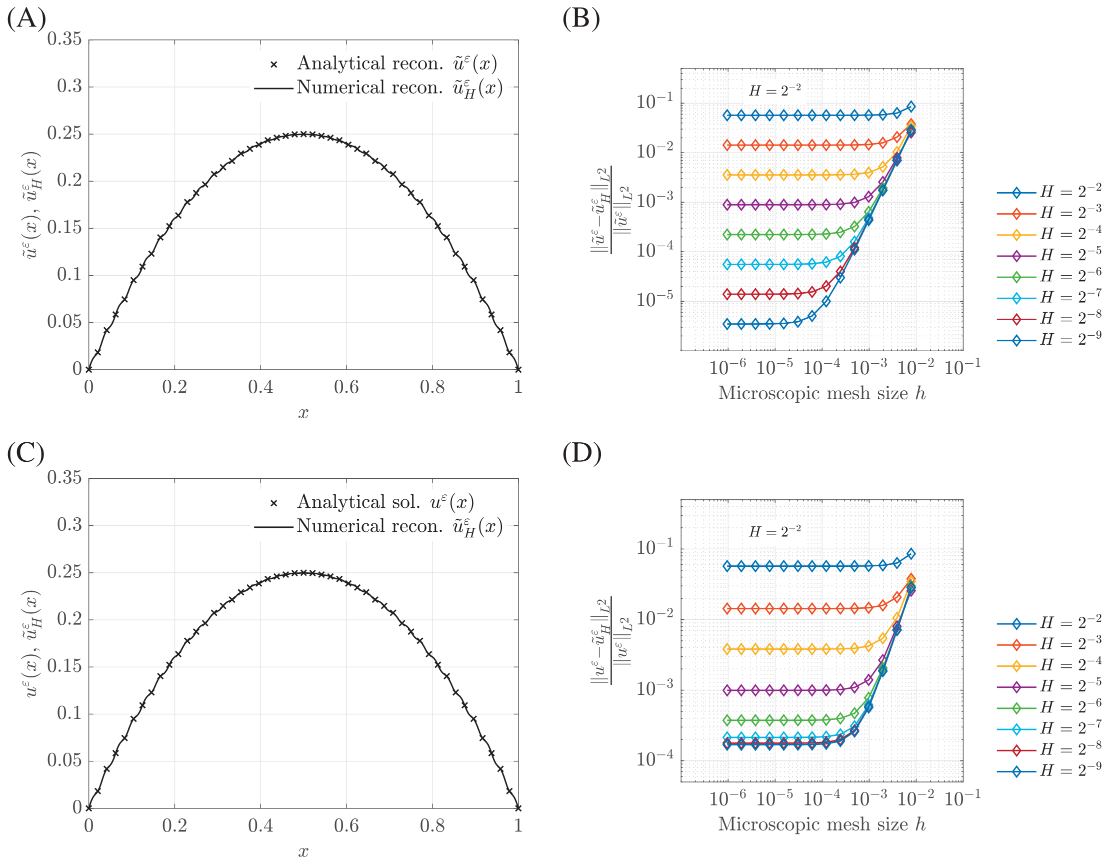

Fig S2.5. Recovered oscillatory solution of the homogenized problem, as given in Eq (S2.12)**, using the proposed multiscale method with a cell size** $\delta = \varepsilon = 2^{-5}$**.** (A) Recovered FE solution $\tilde{u}^{\varepsilon}_H(x)$ in comparison to the analytical recovered solution $\tilde{u}^{\varepsilon}(x)$, see Eq (S2.13), for a macroscopic mesh size $H = \varepsilon$ and a microscopic mesh size $h = \varepsilon/2^{15} \ll \varepsilon$. (B) Evolution of the relative L^2-norm of the error between the analytical solution $\tilde{u}^{\varepsilon}(x)$ and the FE solution $\tilde{u}^{\varepsilon}_H(x)$ for a decreasing microscopic mesh size h. (C) Recovered FE solution $\tilde{u}^{\varepsilon}_H(x)$ in comparison to the oscillatory analytical solution $u^{\varepsilon}(x)$, see Eq (S2.4), for the same discretization, as described in (A). (D) Evolution of the relative L^2-norm of the error between the analytical solution $u^{\varepsilon}(x)$ and the FE solution $\tilde{u}^{\varepsilon}_H(x)$ for a decreasing microscopic mesh size h. Each curve in (B) and (D) corresponds to a constant macroscopic mesh size H. Data used in this figure can be found in the supporting information S1 Data.

The remaining L^2-norm of the error between the analytical oscillatory solution $u^{\varepsilon}(x)$, as

given in Eq (S2.4), and the recovered solution $\tilde{u}^{\varepsilon}(x)$, as computed in Eq (S2.13), is defined as

$$\|u^{\varepsilon}(x)-\tilde{u}^{\varepsilon}(x)\|_{L^2}=\left(\int_0^1 (u^{\varepsilon}(x)-\tilde{u}^{\varepsilon}(x))^2\mathrm{d}x\right)^{1/2}=\frac{\varepsilon^2}{4\pi^2}\sqrt{\frac{3}{2}}. \tag{S2.14}$$

The corresponding relative L^2-norm of the error reads

$$\begin{aligned}\frac{\|u^{\varepsilon}(x)-\tilde{u}^{\varepsilon}(x)\|_{L^2}}{\|u^{\varepsilon}(x)\|_{L^2}}&=\left(\int_0^1 (u^{\varepsilon}(x)-\tilde{u}^{\varepsilon}(x))^2\mathrm{d}x\right)^{1/2}\left(\int_0^1 (u^{\varepsilon}(x))^2\mathrm{d}x\right)^{-1/2} \\ &=\frac{\varepsilon^2}{4\pi^2}\left(\frac{1}{45}+\frac{\varepsilon^2}{16\pi^2}+\frac{25\varepsilon^4}{32\pi^4}\right)^{-1/2}.\end{aligned} \tag{S2.15}$$

For a period length of $\varepsilon = 2^{-5}$, a remaining relative error of $1.66 \cdot 10^{-4}$ is obtained. Fig S2.5 highlights the effects of microscopic and macroscopic mesh refinements on the recovered oscillatory solution. The multiscale FE solution $\tilde{u}^{\varepsilon}_{h}(x)$ is thereby bench-marked against the analytical recovered solution $\tilde{u}^{\varepsilon}(x)$ in Fig S2.5A and S2.5B, and against the analytical oscillatory solution $u^{\varepsilon}(x)$ in Fig S2.5C and S2.5D by means of the relative L^2-norm of their difference. Fig S2.5D clearly shows that the relative macroscopic error converges to the analytically obtained value $1.66 \cdot 10^{-4}$ for a decreasing macroscopic mesh size H.

In conclusion, the performed numerical validation studies revealed that the effective coefficients obtained by the proposed multiscale method are in good agreement with the Monte Carlo simulation results. The proposed multiscale method has significantly smaller computational costs. Therefore, it seems to be a valuable tool to study the impact of various cytoskeletal alterations on the diffusivity within the cytoplasm of a whole eukaryotic cell. However, verification studies have shown that for a perfectly periodic medium the size of the sampling domain, as well as the micro- and macroscopic mesh sizes need to be chosen carefully to obtain good approximations of the effective properties and an accurate homogenized solution. To apply the homogenization concept for periodic media on the cytoskeleton of a eukaryotic cell, we need to extend the concept of homogenization to random structures, i.e. we interpret periodicity as stochastic homogeneity. Thereby, the sampling domain's size needs to be chosen sufficiently large such that a statistically stationary effective coefficient is obtained for randomly chosen sampling domains of the same size within a certain region of the cytoskeleton.

References

1. Novak IL, Kraikivski P, Slepchenko BM. Diffusion in cytoplasm: effects of excluded volume due to internal membranes and cytoskeletal structures. Biophys J. 2009;97:758–767.

2. Novak IL, Gao F, Kraikivski P, Slepchenko BM. Diffusion amid random overlapping obstacles: Similarities, invariants, approximations. J Chem Phys. 2011;134:154104.
