# Supplementary material for: Numerical analysis of the impact of cytoskeletal actin filament density alterations onto the diffusive vesicle-mediated cell transport
Source: PLoS Comput Biol. 2021 May 3;17(5):e1008784. doi: 10.1371/journal.pcbi.1008784 (PMC8130967; doi:10.1371/journal.pcbi.1008784)
Supplement: Attachment — Submitted filename: Reply_to_Reviewer_Nr_2-R1.pdf [file pcbi.1008784.s004.pdf]

Numerical Analysis of the Impact of Cytoskeletal Actin Filament Density Alterations onto the Diffusive Vesicle-Mediated Cell Transport (PCOMPBIOL-D-20-01624)

Authors' Response to the Comments of Reviewer 2:

We would like to sincerely thank the reviewer for the valuable and constructive comments on our paper. We have carefully considered all the comments point-by-point and revised the manuscript accordingly. Below we have re-produced the reviewer's comments followed by our replies in red color. For the sake of clarity, in the revised version of the manuscript the changed text is also marked in red.

In the manuscript entitled, 'Numerical analysis of the impact of cytoskeletal actin filament density alterations onto the diffusive vesicle-mediate cell transport', the authors present a mathematical model and a finite-element formulation for solving the partial differential equations resulting from the model.

There are several issues with the manuscript in its present form that make it hard to evaluate whether it achieves the goal of diffusion of vesicles as a function of actin filament density.

1) The introduction presents a very long summary of many topics in cell biology but doesn't actually outline the most recent literature on actin organization in cells or passive transport of vesicles. There is no mention of vesicle sizes or components. Furthermore, the authors have not consulted the recent advances in the literature on actin organization in cells (for example: https://www.nature.com/articles/ncb2044 and https://www.cell.com/structure/pdf/S0969-2126(16)30086-7.pdf). Indeed, a paper on actin organization without the mention of papers by stalwarts in the field such as Thomas Pollard is rather surprising. Furthermore, the actin organization is not the same across the different parts of the cells and actin is highly regulated by many actin binding proteins. Therefore, as written, it is not clear that the authors are actually modeling an actin network.
Thank you! We have worked through the suggested references on actin filament organization and considered them among others in the revised version of this manuscript, see Sections 1 and 2.1. Furthermore, we added information regarding typical vesicle sizes in Section 1.
As identified correctly by the reviewer, there are numerous different actin binding proteins, such as Arp2/3, α-actinin, fimbrin, filamin, etc. involved in the network formation, and the organization of the actin filaments varies significantly inside the cell, e.g., the dense meshwork in the cell cortex, the stress fibers, or the parallel actin bundles in the filopodium, which we have already mentioned in Section 1. Nevertheless, we emphasize this limitation of the study once again in Section 4.
However, the major objective of the current study is to introduce a fast numerical method which allows to computationally investigate the effect of different actin filament densities – due to growth and remodeling – onto the diffusive vesicle transport inside the whole cell considering some of the fine scale details of the cytoskeleton, on the basis of a 2D problem. In a follow up study, the implemented multiscale finite element method will be applied to an improved 3D model of the cytoskeleton considering the microtubules and intermediate filaments based on electron microscopy investigations yielding more detailed structural information.

2) I read the paper by Miendl et al. 2017 on which the current paper is built on very carefully. In that study, they use nanoparticles and it is not clear that in the current study that nanoparticles are modeled. Nanoparticles and vesicles are used interchangeably (they are not). More importantly, the data in Miendl et al for actin is not at a resolution to reconstruct an actin network. Also, the data is only in 2D and therefore, it is not clear how the authors built a 3D meshwork of actin. Clearly the assumptions underlying these steps are important for building the 3D network. The resulting model does not represent the features of actin. A specific

example of this is that the network constructed here has intersection angles of 46.39 degrees, while actin branch angles are known to be approximately 70 degrees (again many references that have established this point). These two points essentially lead me to the conclusion that the biological assumptions underlying this work are flawed.
Thanks! As correctly identified by the reviewer, the limited resolution of the confocal microscopy images produced by Meindl et al. (2017) does not allow the fine details of the actin filament network to be reconstructed, such as the structure of individual filaments and their branching behavior. However, an accurate mapping of these features is the key to determine more realistic diffusion properties. Therefore, for a specific macroscopic point, a generic 3D microscopic actin filament network was created based on the developed 2D macroscopic actin density models (obtained for each slice of the z-stack, see Section 2.1.1) combined with published data regarding the in vitro spatial actin filament structure (i.e. orientation distribution, branching properties, etc.), as described in detail in Section 2.1.2. We have now tried to make the utilized modeling approach more precise by adding a paragraph in Section 2.1.
We appreciate the constructive comments regarding the branching angles. We have now considered that in the revised version. Furthermore, we have repeated some of the simulations performed in Section 3.3 in the original manuscript. Thereby, we allowed Arp2/3-specific branching angles between 65 and 75 degrees and created several representative actin filament network models having specific filament densities, and a dimension of (1x1x1) µm, see Fig. 2(a). For each network model, mean branching angles ranging between 68.15° and 72.47° were obtained. Afterwards, we quantified the inaccessible area fraction and the effective diffusion coefficients for a single horizontal cut through each unit cube at z=0.5µm, see Fig. 2(b), using the proposed multiscale FE method. Since we analyze the effective diffusion coefficients only for a 2D sampling domain cut out of the 3D network, the changes of the branching angles had neglectable effects on the results (max. deviation of 0.15%), as presented in the original manuscript, see especially Table 1, Fig. 10 and the parameters of the power law. Note that the estimated parameters µ and φ_c were not influenced at all due to the altered branching angles. However, for a follow up study in 3D on the inaccessible volumes, the influence of the branching angles will be significant. Therefore, we would like to thank the reviewer for this valuable remark. Please note that we did not change the corresponding figures in the revised version of the manuscript since the alterations of the results are marginal and barely visible.
We replaced the terms "(nano-)particle" and "tracer" by the term "vesicle" throughout the manuscript, since we are focusing on the vesicle-mediated cell transport in this study. We used only the data regarding the actin filament density from the work by Meindl et al. (2017).

3) The model that the authors develop for the vesicle concentration is a diffusion model. I have the following issues with the model.
    a. What does vesicle concentration mean? Number of vesicles per unit volume? are vesicles present in a large enough density that a concentration can be defined for them? In a cube of 1 cubic micron, how many such vesicles are there?
    By vesicle concentration we mean the number of vesicles per 1 µm³. In the diffusion model, we normalize the vesicle concentration u(x) by the maximum possible number of vesicles per µm³ such that $0 \leq u(x) \leq 1$. *Busy cells are often filled with thousands of traveling vesicles. In the case of ribbon synapses, e.g., the number of vesicles per µm³ with an averaged diameter of 46 nm is location-dependent and ranges between 212±69 and 524±84, with higher values closer to the cell membrane (Holt et al., 2004). This information has now been added to the manuscript in Section 1. Thus, in the authors' opinion, vesicles are present in a large enough density such that a concentration can be defined for them.*

b. What do the boundary conditions translate to in the physical domain? Are the boundaries representing the plasma membrane? The organelle membrane? Or the filaments? Do the filaments provide a physical barrier for diffusion?
In Sections 2.2 and 2.3 of the revised manuscript, we have now provided a (hopefully) much clearer description of the described mathematical model's physical meaning. We define the physical interpretation of the Dirichlet boundary conditions (i.e. the intracellular vesicle concentration along the cell membrane), the Neumann boundary conditions (i.e. the flux of vesicles through the cell membrane), and make clear that the vesicles are not able to penetrate the cytoskeletal obstacles. Therefore, the discretized and analyzed sampling domains K_δ*, as illustrated in Fig. 4(c), contain holes – the inaccessible domains due to the obstacles and the finite size of the vesicles.*

c. It is also noteworthy that the authors do not reference the works by Paul Bressloff and other colleagues who have developed extensive models on diffusion of particles and vesicles in crowded spaces.
Indeed, the work of Paul Bressloff is outstanding and some of his contributions have now been cited in the revised version of the manuscript.

d. Does the vesicle size not matter? There is no consideration given to this factor. What about thermal fluctuations in the cellular environment?
In Section 3.1 of the revised manuscript, we have now numerically investigated and discussed the impact of the vesicle radius concerning the inaccessible area fraction for different actin filament densities and, consequently, the diffusion coefficient. Furthermore, we have clarify that all simulation results correspond to a homogeneously distributed constant temperature of 37°C inside the cell. As pointed out by the reviewer, any deviations from this value have a direct impact on the diffusivity; see, e.g., the Stokes-Einstein relation, which is used in the study to estimate the in vitro diffusion coefficient.

It appears that the authors developed a mathematical and finite element model for a diffusive process, which in and of itself is fine. However, this model has very little to do with the biological process the authors claim to study. If the authors wish to write a paper on the mathematical aspects of their model, then they should do so and consider sending it to a suitable journal. If the authors wish to address a major problem in cell biology, then the details of the biological process are important and more than one set of experiments should be taken into account. Furthermore, the model should be validated against data that is already published and then generate experimentally testable predictions. Right now, this manuscript does neither.
Once again, we would like to sincerely thank the reviewer for the valuable and constructive comments on our paper. We have carefully considered all the comments point-by-point and revised the manuscript.
However, we would like to emphasize that the main objective of this paper was to introduce a very efficient numerical multiscale FE tool for modeling the diffusive part of the vesicle-mediated, which is able to consider the fine details of the cytoskeletal filament network. It allows to analyze the impact of actin filament density alterations onto the diffusion coefficient and, consequently, the hypothetical traveling time of vesicles throughout the cells. Of course, diffusion itself is not the transport of choice and vesicle might not diffuse 20 µm in vitro or in vivo. Nevertheless, the current study shows how alterations of the actin filament network density – due to growth and remodeling mechanisms triggered by mechanotransduction – affect the local diffusion coefficients and, therefore, the transport time using the proposed multiscale FE method. In the authors' opinion, this study utilizes a new and efficient computational method to analyze an open and interesting issue in cell biology, and hence would fit to the journal's scope.

In the revised version of this manuscript we have now added new simulation results and have thoroughly discussed the impact and importance of investigating the effect of actin filament density alterations with respect to the vesicle-mediate cell transport. Note that we compare the obtained local diffusion coefficients with values obtained from experimental studies, and provide an idea how the findings of this current study can be validated by future experiments in a controlled environment.

References
[1] Holt et al., 2004. High mobility of vesicles supports continuous exocytosis at a ribbon synapse. Curr. Biol. 14: 173–183.
